# Impact and Perceived Value of iGeriCare e-Learning Among Dementia Care Partners and Others: Pilot Evaluation Using the IAM4all Questionnaire

**DOI:** 10.2196/40357

**Published:** 2022-12-22

**Authors:** Andrew F Scott, Stephanie Ayers, Pierre Pluye, Roland Grad, Richard Sztramko, Sharon Marr, Alexandra Papaioannou, Sandra Clark, Patricia Gerantonis, Anthony J Levinson

**Affiliations:** 1 Faculty of Health Sciences McMaster University Hamilton, ON Canada; 2 Division of e-Learning Innovation McMaster University Hamilton, ON Canada; 3 Department of Family Medicine McGill University Montreal, QC Canada; 4 Department of Medicine McMaster University Hamilton, ON Canada

**Keywords:** dementia, caregiver, web-based education, internet, consumer health information, feedback, perception, survey, questionnaire, patient education, health education, care partner, caregiving, spousal care, informal care, Alzheimer, cognitive impairment, cognitively impaired, Lewy body, gerontology, geriatric

## Abstract

**Background:**

Care partners of people living with dementia may benefit from web-based education. We developed iGeriCare, an award-winning internet-based platform with 12 multimedia e-learning lessons about dementia.

**Objective:**

Our objective was to evaluate users’ perceptions of impact.

**Methods:**

From March 17, 2021 to May 16, 2022, data were collected upon lesson completion. We used the content-validated Information Assessment Method for all (IAM4all) for patients and the public adapted for dementia care partners. The IAM4all questionnaire assesses outcomes of web-based consumer health information. Responses were collected using SurveyMonkey, and data were analyzed using IBM SPSS Statistics (version 28).

**Results:**

A total of 409 responses were collected, with 389 (95.1%) survey respondents completing the survey. Of 409 respondents, 179 (43.8%) identified as a family or friend care partner, 84 (20.5%) identified as an individual concerned they may have mild cognitive impairment or dementia, 380 (92.9%) identified the lesson as relevant or very relevant, and 403 (98.5%) understood the lesson well or very well. Over half of respondents felt they were motivated to learn more, they were taught something new, or they felt validated in what they do, while some felt reassured or felt that the lesson refreshed their memory. Of 409 respondents, 401 (98%) said they would use the information, in particular, to better understand something, discuss the information with someone else, do things differently, or do something.

**Conclusions:**

Users identified iGeriCare as relevant and beneficial and said that they would use the information. To our knowledge, this is the first time the IAM4all questionnaire has been used to assess patient and caregiver feedback on internet-based dementia education resources. A randomized controlled trial to study feasibility and impact on caregiver knowledge, self-efficacy, and burden is in progress.

## Introduction

Informal family care for people living with dementia is important, as 61% of Canadian seniors with dementia live at home [1]. Those living with dementia are often supported by informal care partners who each provide an average of 26 hours of care per week [1,2]. Care partners of people living with dementia may benefit from web-based education to help them develop knowledge and skills to support themselves and the person to whom they are providing care [2,3]. Caregiver education is most commonly delivered through face-to-face interactions during a clinical visit [4]. This can cause issues for clinicians because of time and resource constraints. Access to in-person dementia education resources for caregivers may also be very limited in some rural communities. Internet-based caregiver education has the potential to address some of these challenges and allow dementia caregivers and others to access educational materials at any time and at their own pace.

Although a systematic review performed by Moore and colleagues [5] reported inconclusive evidence supporting the effectiveness of educational interventions focused specifically on dementia progression on dementia caregivers’ knowledge and mental health, individual studies have shown favorable outcomes. The heterogeneity in format, length, instructional design methods, and content of interventions may play a role in some of the inconsistent findings of systematic reviews. This suggests that it may be important to compare educational interventions with more clearly defined instructional designs [6], especially those that follow best practices for web-based learning environments [7]. With the benefits of internet-based interventions on mental health for care partners of adults living with a chronic condition being shown by Sherifali and colleagues [8] and the reported need for high-quality, trusted internet resources for dementia caregivers, it is worthwhile to continue to study high-quality educational interventions that touch on a variety of topics and take into consideration the needs of caregivers [2,3].

For these reasons, we developed iGeriCare, an internet-based platform with 12 multimedia e-learning lessons about dementia. iGeriCare lessons cover a wide range of dementia-related topics, including stages of dementia, treatment options, and caregiver wellness, among others. The lessons were created using best practices in evidence-based instructional design for e-learning [9,10] by experts in dementia care with input from care partners, winning the first-place e-Learning Excellence Award at the 2021 European Conference on e-Learning [11-13]. Since launching in July 2018, iGeriCare has had over 182,000 unique users and over 258,000 user sessions. Lessons are multimedia content with images, audio narration, authentic case studies and examples, as well as some interactivity such as review quizzes, approximately 20 minutes in duration each (Multimedia Appendix 1). All iGeriCare lessons are publicly available and free to access. Our objective was to evaluate users’ impressions of the multimedia lessons, in particular, their perceived relevance, understandability, usefulness, and expected benefit.

## Methods

### Overview

From March 17, 2021, to May 16, 2022, anonymous data were collected from any person who completed an iGeriCare lesson and opted to fill out a postlesson survey. A total of 12 lessons were available, and the survey was presented after each lesson. Respondents could access iGeriCare in a variety of ways, including a recommendation from a health care professional or teacher, clicking on a link from a social media advertisement or from another website, doing an internet search, receiving the link from family or friends, or being an existing email subscriber to the iGeriCare newsletter campaign.

After lesson completion, participants were asked to rate the lesson on the Net Promoter Score (NPS) scale. NPS is a management tool that can be used to gauge customer satisfaction [14-16]. Using an 11-point scale, the NPS asks respondents their “likelihood to recommend” a product or service based on their experience. Participants were then invited to take the postlesson survey through a link. The 11-question postlesson survey was developed using the Information Assessment Method for all (IAM4all) questionnaire adapted for dementia care partners. The IAM4all is a content-validated questionnaire designed to collect feedback from health information consumers (eg, the public or patients) about resources, and it is structured by four levels of outcomes, including situational relevance, cognitive impact, information use, and health benefits [17]. The survey incorporated conditional logic that allowed respondents to skip some questions based on their responses (eg, if a respondent answered “no” to “Will you use the information from this lesson?” they would automatically skip the question “Please tell us how you will use this information”; Multimedia Appendix 2).

SurveyMonkey was used to collect survey responses, and all data were transferred into IBM SPSS Statistics (version 28; IBM Corp) for statistical analyses. All data from respondents—including those who only partially completed the survey—were included in the data analysis, and the survey data for all lessons were pooled. For analyses of differences in responses by respondent type or role, the Independent-Samples Kruskal-Wallis test with post hoc pairwise comparisons adjusted by the Bonferroni correction for multiple tests was used.

### Ethics Approval

The Hamilton Integrated Research Ethics Board reviewed the study protocol and granted exemption from full review per their review process, as this was considered a quality improvement initiative.

## Results

### Data Trends and Respondent Characteristics

During the 14-month data collection period, there were a total of 2915 iGeriCare lesson completions. Of those, 409 respondents (14% of lesson completers) started an IAM4all survey. Of 409 respondents, 389 (95.1%) who started the postlesson survey completed it, with partial completion for the remaining 20 respondents. Average time spent for survey completion was 3 minutes and 11 seconds. The most commonly completed lessons for survey respondents were the following: “What is dementia?” with 153 of 409 (37.4%) lesson completions; “What is mild cognitive impairment?” with 71 of 409 (17.4%) lesson completions; and “How to promote brain health” completed by 46 of 409 (11.2%) respondents.

A total of 179 of 409 (43.8%) respondents self-identified as a family or friend care partner of a person living with dementia. Those concerned they may have cognitive impairment made up 84 of 409 (20.5%) respondents, with a further 37 (9%) having a diagnosis of mild cognitive impairment (MCI) or dementia. The smallest groups were health students (35/409, 8.6%) and health care professionals (41/409, 10%); 33 of 409 (8.1%) self-identified as “other.” Respondents arrived at the iGeriCare site in various ways, with the most common being the recommendation of a health care professional.

### IAM4all Domains

Responses were extremely positive with respect to the relevance, understandability, information use, and benefits of the lessons. Of the 409 respondents, 263 (64.3%) perceived the lesson as very relevant, and 117 (28.6%) perceived the lesson as relevant, compared to 27 (6.6%) who said it was somewhat relevant and only 2 (0.5%) who found it not very relevant. 306 of 409 (74.8%) respondents understood the lesson very well, and 97 (23.7%) understood the lesson well; only 6 (1.5%) said they understood it poorly, and no one said they understood it very poorly. Similarly, 401 of 409 (98%) said they would use the information from the lesson, and 378 of 405 (93.3%) expected to benefit from it.

Participants identified a range of thoughts about the lesson they reviewed, with over half stating that it taught them something new, motivated them to learn more, or validated what they were already doing; 194 of 409 (47.4%) respondents found the lessons reassuring, and 154 of 409 (37.7%) said it refreshed their memory. For those who said they would use the information from the lesson (n=396, as 13 respondents skipped this item), 281 of 396 (71%) respondents said they would use it to better understand something; 206 of 396 (52%) said they would use it to discuss with someone else; 134 of 396 (33.8%) said they would use the information to do things differently; and 121 of 396 (30.6%) said they would use it to do something.

Nearly half of 372 respondents identified that the lesson would benefit them by improving their health or well-being or that of the person they care for (Table 1); 177 of 372 (47.6%) said it would help them handle a problem or the worsening of a problem; 120 of 372 (32.3%) said it would prevent a problem; 160 of 372 (43%) felt they would be less worried; and 105 of 372 (28.2%) said it would benefit them by helping them to decide something for someone else.

**Table 1:** Selected summary of Information Assessment Method for all (IAM4all) questions and participant responses.

Variables			Responses, n (%)
**What did you think about this lesson? (N=409)**			
	Motivated me to learn	236 (57.7)	
	Learned something new	219 (53.5)	
	Validated what I do	218 (53.3)	
	Reassured me	194 (47.4)	
	Refreshed my memory	154 (37.7)	
	Did not like this lesson	5 (1.2)	
**I will use this information to (N=396)**			
	Better understand something	281 (71)	
	Discuss with someone	206 (52)	
	Do things differently	134 (33.8)	
	Do something	121 (30.6)	
	Other	42 (10.6)	
**This information will help me to (N=372)**			
	Improve my health	181 (48.7)	
	Improve the health of someone I care for	177 (47.6)	
	Handle a problem	177 (47.6)	
	Be less worried	160 (43)	
	Prevent a problem	120 (32.3)	
	Decide something for someone else	105 (28.2)	
	Other	42 (11.3)	

### Response Differences Between Participant Types

Data collected from IAM4all questions were then analyzed by participant type, that is, family or friend care partner, a respondent concerned they might have MCI or dementia, someone diagnosed with MCI or dementia, a student or trainee, a health care provider, or other participant types. Perceived relevance was significantly higher among family or friend care partners compared to people concerned with possibly having MCI or dementia [*H*(5)=14.533; *P*=.01]. Those with a diagnosis or concerned they might have MCI or dementia were significantly less likely to understand a lesson “very well” compared to the other groups [*H*(5)=41.762; *P*<.001], although 117 of those 121 (96.7%) respondents still reported understanding the information “well” or “very well,” with only 4 reporting they understood it “poorly.” Analyses of other survey data did not show any statistically significant differences by respondent role.

## Discussion

### Principal Findings

The purpose of this work was to evaluate iGeriCare users’ impressions of the multimedia lessons, including their perceived relevance, understandability, usefulness, and expected benefit, using the IAM4all survey. Respondents to the survey identified the iGeriCare lessons as relevant and beneficial, and they said they would use the information. Responses were relatively consistent among different types of participants.

These findings are of interest for two main reasons: first, although the iGeriCare site was primarily designed for family care partners, less than half of respondents identified as a family or friend care partner, implying that the iGeriCare site has appeal to many different audiences, including those concerned they may have a cognitive impairment, those who already have MCI or dementia, and even health care professionals and trainees. Second, the similarity in responses despite the varied audience implies that the iGeriCare lessons are not only appealing to a broader demographic but also relevant and valuable to a wider audience. Although 380 of 409 (92.9%) respondents perceived the lessons as either relevant or very relevant, the finding that the perceived relevance was greater for family and friend care partners compared to those concerned with having MCI or dementia is consistent with the initial design and intent of the site, as the scenarios presented within the e-learning lessons use family care partners.

Furthermore, even though 403 of 409 (98.5%) of respondents understood the lesson well or very well, those with a diagnosis or concerned they might have MCI or dementia were significantly less likely to understand a lesson “very well” compared to other respondent types. With the iGeriCare site having been co-designed with family care partners and not specifically designed for those with cognitive impairment, this finding seems reasonable and highlights a need for similar resources with lessons and scenarios tailored to individuals with these concerns or diagnoses.

### Comparison to Prior Work

IAM4all questions measure participants’ perceived relevance, intention to use information, and expected benefit. The high percentage of respondents stating that they would use the information and expect benefits is consistent with the NPS ratings of iGeriCare lessons and comparable to IAM4all feedback on a high-quality parenting website [18]. The percentage of ‘symbolic use’—referring to those that would use the information to discuss with someone else—was higher on iGeriCare, consistent with promotion primarily to family or friend care partners and the fact that care partners made up the largest respondent type for this survey. This likely represents a mix of different people that respondents will discuss the information with, including the person they are caring for, other family or friend care partners, and their health care providers.

Respondents noted that the information in the lessons motivated them to learn more, taught them something new or refreshed their memory, validated their actions, and provided reassurance. This suggests that iGeriCare lessons are helpful in transferring knowledge and improving confidence of dementia caregivers. Considering four IAM4all constructs, ‘conceptual use’ (eg, better understanding) was most frequently noted, followed by ‘symbolic use,’ ‘instrumental use’ (ie, doing things differently), and ‘legitimating use’ (ie, doing something) [19,20]. These constructs within the IAM4all shed a different light on how dementia care partners and others interact with information resources. Previous systematic reviews have shown the impact of internet-based interventions on care partners’ mental health but have rarely described specific uses of information or perceived benefits using a content-validated tool [8,21].

Pluye and colleagues [17] highlight three ways in which individuals seek health-based information on the internet: professionally mediated (ie, provided by a clinician or librarian), direct access (ie, individual search), and peer mediated (ie, recommendations from family or friends and social media). Respondents in our study arrived at the iGeriCare lessons through all three of these ways and, irrespective of how they arrived at the site, the educational content was highly valued.

### Limitations

There are several limitations to this work. First, there is a potential selection bias, as data are from only those users who both completed a lesson and opted to fill out the additional IAM4all questionnaire. Those users who completed a lesson may be more likely to be positively predisposed to the lesson’s relevance, understandability, usefulness, and perceived benefit. However, NPS ratings between respondents and other users of iGeriCare are similar, suggesting that the participants may be comparable to iGeriCare users more broadly. Additionally, despite this potential selection bias, users who completed lessons were the desired target population for the postlesson surveys, since they had engaged with the complete intervention as intended. Nonetheless, future work should consider including a prompt asking respondents who abandon a lesson why they have opted to leave the lesson before completion. Since users can leave and come back to iGeriCare lessons at any time, an option of ‘I intend to finish this lesson later’ should be included. Second, the same individual may have filled out the IAM4all multiple times for different lessons. Due to the anonymity of the site and survey, we had a limited idea of how many respondents completed the IAM4all for different lessons as well as limited information about participant demographics. However, this was part of an intentional design to facilitate easy access to iGeriCare lessons, rather than requiring users to create a free account. Furthermore, it is reasonable to assume that the same respondent may react differently to each lesson, eliminating the need to identify unique respondents between lessons. For these reasons, future work should continue to remove as many barriers as possible to educational interventions, even if this may impact the extent of research data about participants.

### Implications and Future Directions

To our knowledge, this is the first time the IAM4all has been used to assess dementia education resources specifically. It is also the first time that the questionnaire has been used to evaluate instructionally designed, multimedia e-learning, rather than predominantly text-based formats of health information (eg, web pages and email-based content). The questionnaire has helped to better delineate care partners’ and others’ perspectives on the value and perceived impact of the iGeriCare lessons.

More work is needed to determine the effectiveness of web-based dementia education for care partners on other validated outcome measures. A randomized controlled trial to study impact on care partner knowledge, self-efficacy, and sense of burden is in progress (Clinicaltrials.gov identifier NCT05114187).

## Appendix

Multimedia Appendix 1 Screenshot from an iGeriCare lesson.

Multimedia Appendix 2 A sample of the IAM4all questions in the postlesson survey.

## Abbreviations

IAM4all: Information Assessment Method for all
MCI: mild cognitive impairment
NPS: net promoter score

## Data Availability

The data sets generated and analyzed during this study are available from the corresponding author on reasonable request.
