# Supplementary figures and images for: Impact and Perceived Value of iGeriCare e-Learning Among Dementia Care Partners and Others: Pilot Evaluation Using the IAM4all Questionnaire
Source: JMIR Aging. 2022 Dec 22;5(4):e40357. doi: 10.2196/40357 (PMC9816950; doi:10.2196/40357)

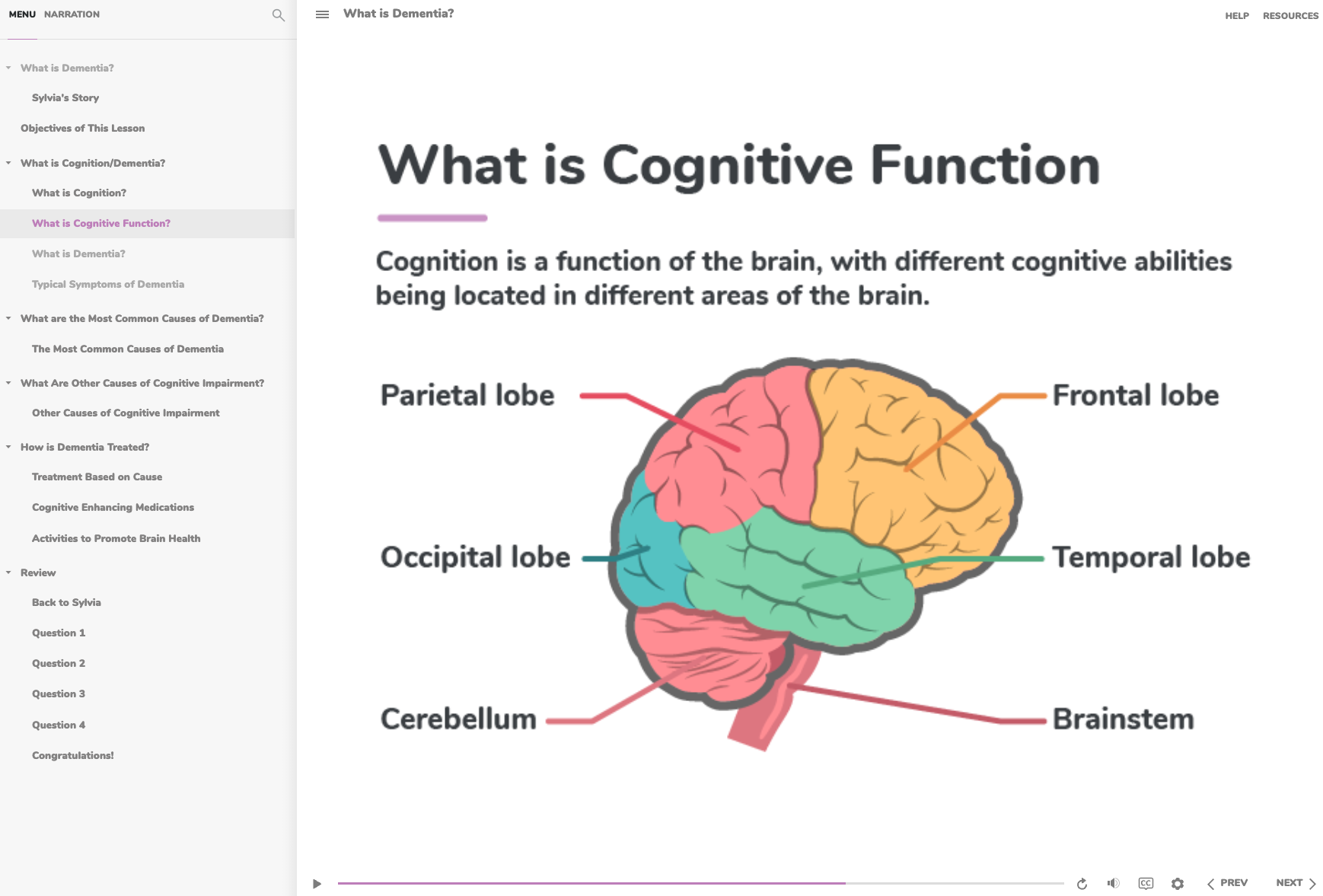

Supplement: Multimedia Appendix 1 [file aging_v5i4e40357_app1.png]

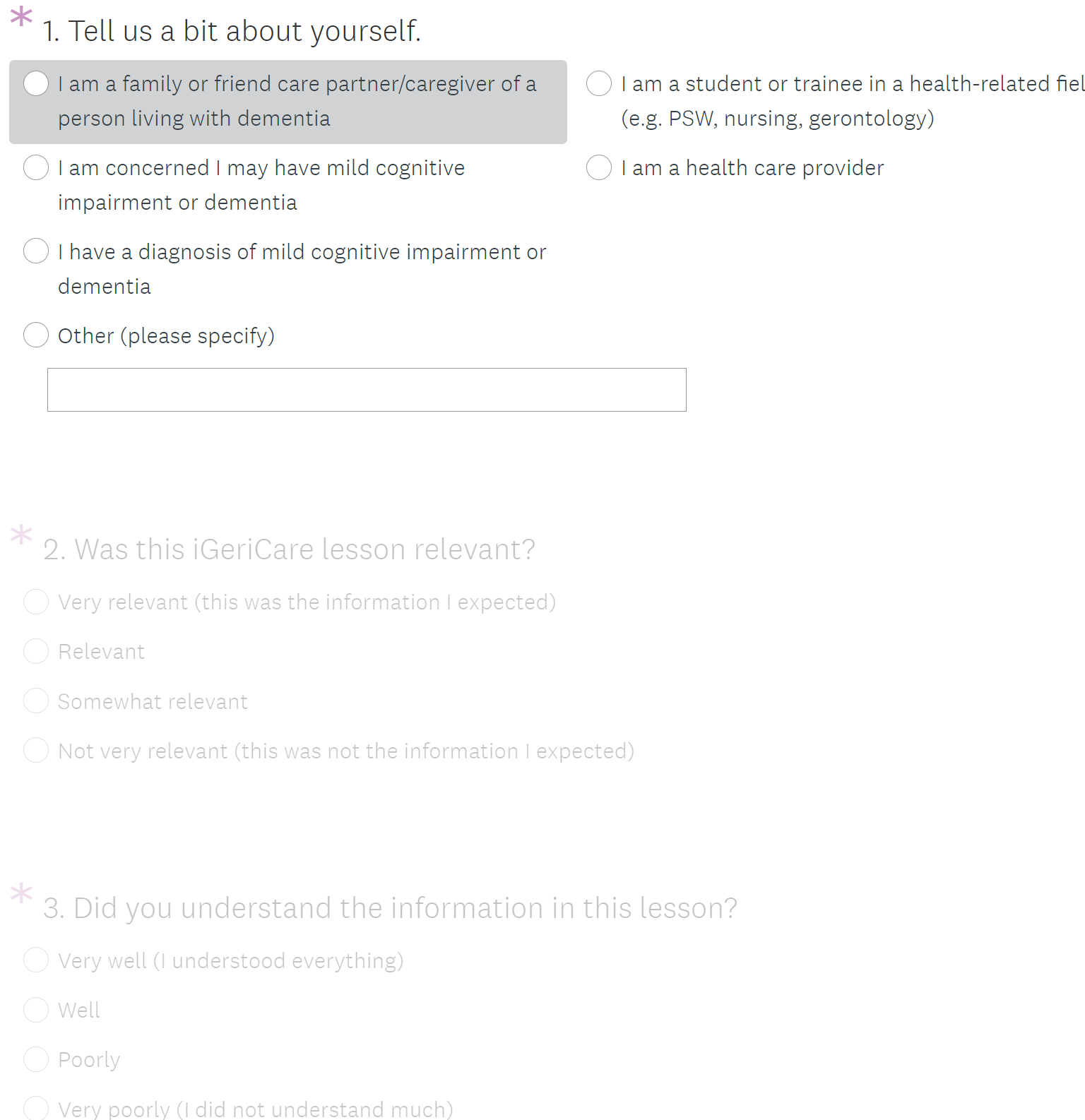

Supplement: Multimedia Appendix 2 [file aging_v5i4e40357_app2.png]
